# Membrane Potential Measurements of Isolated Neurons Using a Voltage-Sensitive Dye

**DOI:** 10.1371/journal.pone.0058260

**Published:** 2013-03-13

**Authors:** Richard Fairless, Andreas Beck, Mykola Kravchenko, Sarah K. Williams, Ulrich Wissenbach, Ricarda Diem, Adolfo Cavalié

**Affiliations:** 1 Department of Neuro-Oncology, University Clinic Heidelberg, Heidelberg, Germany; 2 Department of Neurology, University of Saarland, Homburg/Saar, Germany; 3 Department of Pharmacology and Toxicology, University of Saarland, Homburg/Saar, Germany; University G. D’Annunzio, Italy

## Abstract

The ability to monitor changes in membrane potential is a useful tool for studying neuronal function, but there are only limited options available at present. Here, we have investigated the potential of a commercially available FLIPR membrane potential (FMP) dye, developed originally for high throughput screening using a plate reader, for imaging the membrane potential of cultured cells using an epifluorescence-based single cell imaging system. We found that the properties of the FMP dye make it highly suitable for such imaging since 1) its fluorescence displayed a high signal-to-noise ratio, 2) robust signals meant only minimal exposure times of around 5 ms were necessary, and 3) bidirectional changes in fluorescence were detectable resulting from hyper- or depolarising conditions, reaching equilibrium with a time constant of 4–8 s. Measurements were possible independently of whether membrane potential changes were induced by voltage clamping, or manipulating the ionic distribution of either Na^+^ or K^+^. Since FMP behaves as a charged molecule which accumulates in the cytosol, equations based on the Boltzmann distribution were developed determining that the apparent charge of FMP which represents a measure of the voltage sensitivity of the dye, is between −0.62 and −0.72. Finally, we demonstrated that FMP is suitable for use in a variety of neuronal cell types and detects membrane potential changes arising from spontaneous firing of action potentials and through stimulation with a variety of excitatory and inhibitory neurotransmitters.

## Introduction

The imaging of isolated neurons through various microscopic techniques is an essential part of elucidating neuronal function at the cellular level. In order to monitor various aspects of neuronal function in real time, an array of dyes has been developed which can be applied extracellularly. For instance, the intracellular Ca^2+^ concentrations can be monitored with FURA-2 [1], changes in intracellular Na^+^ levels through use of SBFI [2] and vesicle turnover using FM styryl dyes [3]. However, they do not cover the most important function of the neuron, the ability to communicate through the generation of action potentials. Today, genetically-encoded voltage sensors are available which can be used to monitor changes in membrane potential *in vivo* as well as in multicellular preparations such as brain slices [4]. Similar to FURA-2 and related compounds, voltage-sensitive dyes (VSDs) have recently been developed for monitoring membrane potential changes by taking advantage of a wide variety of voltage-sensing mechanisms [5], [6], [7]. With the exception of JPW-114 [8], VSDs are usually applied extracellulary. Recently, a VSD was made commercially available to investigate cell membrane potential but its current use has mainly been limited to the fluorometric imaging plate reader (FLIPR), where it is used for high-throughput screening [9], [10], [11], [12]. Although high-throughput screening systems require robust fluorescence signals, little else is known about the voltage sensitivity and kinetics of the dye or about possible interactions with neurotransmitters that are used in studies of neuronal function.

Here, we have investigated the potential of the FLIPR membrane potential (FMP) dye for measuring membrane potential changes in neurons using a common single cell imaging system. Firstly, we demonstrate that FMP accumulates in the cytosol and provides a high signal-to-noise ratio with 3 times more intense fluorescence in neurons over the background fluorescence of feeder layers. Secondly, due to the direct proportionality between FMP signals and the degree of depolarisation or hyperpolarisation, precise quantification of changes in membrane potential can be achieved using FMP. This was demonstrated both by modulating external K^+^ and through direct voltage-clamping of the cell membrane. Thirdly, we provide evidence that the dye is measuring membrane potential changes independently of the ion type that determines the change. Finally, we demonstrate that it is suitable for use in a variety of neuronal subtypes, and can detect membrane potential changes arising both from spontaneous firing of action potentials, and through stimulation with a variety of excitatory and inhibitory neurotransmitters.

Thus, we believe that this dye has great potential for imaging of isolated neurons and, due to the ease of application, FMP imaging will rapidly become an established protocol for measuring membrane potential as FURA-2 imaging is for measurement of internal calcium.

## Materials and Methods

### Ethics Statement

This study was carried out in strict accordance with guidelines of the European Union on the protection of animals used for scientific purposes (2010/63/EU). The protocols and procedures involving animals were approved by the Animal Welfare Office of the University of Saarland and by the Central Veterinary Office of Saarland (H-1 2.4.3.5).

### Cell Preparation and Culture

#### HEK and HEK-TRPM8 cells

Human embryonic kidney (HEK) cells were grown at 37°C with 5% CO_2_ in DMEM (Invitrogen, Darmstadt, Germany) supplemented with 10% foetal calf serum. The HEK cell-line stably expressing human TRPM8 (TRPM8-HEK) has been described previously [13] and was cultured in the presence of G418 (500 µg/ml). Naïve HEK and TRPM8-HEK cells were plated on poly-L-lysine-coated coverslips and cultured for 2–3 days before recordings.

#### RGCs

Primary retinal ganglion cells (RGCs) were obtained from 6 to 8 days old brown Norway rats according to a two-step immunopanning protocol described previously [14]. Cells were seeded onto poly-D-lysine-coated coverslips and cultured in serum-free Neurobasal medium (Invitrogen) containing glutamine, cysteine, B27 supplement, sodium pyruvate, triiodothyronine and sato’s medium (transferrin, bovine serum albumin, progesterone, putrescine and sodium selenite) supplemented with forskolin (4.1 µg/ml), ciliary neurotrophic factor (10 µg/ml), brain-derived neurotrophic factor (50 µg/ml) and insulin (5 µg/ml) at 37°C in 5% CO_2_. Imaging experiments were carried out 5–7 days after plating.

#### Hippocampal neurons

Primary hippocampal neurons were prepared and cultured as previously described [15]. Briefly, cells were dissociated from the hippocampi of newborn mice (C57Bl/6 strain) in calcium- and magnesium-free Hanks’ balanced salt solution (Invitrogen) by 20 min incubation with 0.25% trypsin, and plated on poly-D-lysine-coated coverslips. Cells were maintained at 37°C with 5% CO_2_ in DMEM supplemented with 10% foetal calf serum, antibiotics (100 U/ml penicillin, 100 µg/ml streptomycin) and 2 mM glutamine for 24 hours, before exchanging media for Neurobasal medium including B27 supplement, antibiotics and 0.5 mM glutamine. Cultures were grown for 7–12 days before imaging.

#### DRGs

Dorsal root ganglion (DRG) neurons were dissociated and cultured essentially as previously reported [16]. Adult mice (C57Bl/6 strain) were anesthetized by intraperitoneal application of a urethane solution (2 mg urethane/g weight). DRGs were quickly removed and incubated in trypsin type 1 (1 mg/ml medium) and collagenase type II (4 mg/ml medium) for 20 min. Cells were resuspended in DMEM supplemented with 10% foetal calf serum and antibiotics (100 U/ml penicillin and 10 µg/ml streptomycin). Neurons were plated onto poly-L-lysine coated glass coverslips and cultured at 37°C with 5% CO_2_ for 7–12 days before imaging.

#### Cortical and septal neurons

The whole septum and rinds of the frontal cortex were dissected from early postnatal mouse brain (C57Bl/6 strain) basically as previously described [17], [18]. Subsequently, the dissected cortical rinds were digested and the cell suspensions were cultured following the same procedure used for hippocampal neurons. Imaging experiments were performed on 5–7 days old cultures.

### Recording Solutions

We used either a Hanks’ balanced salt solution (HBSS, Molecular Devices) or a basic solution (BS) with various K^+^ and Na^+^ concentrations for imaging experiments. HBSS was buffered with 20 mM HEPES (pH 7.4) and contained 1.3 mM CaCl_2_, 5.4 mM KCl, 0.4 mM KH_2_PO_4_, 0.5 mM MgCl_2_, 0.4 mM MgSO_4_, 136.9 mM NaCl, 0.3 mM Na_2_HPO_4_, 4.2 mM NaHCO_3_ and 5.5 mM glucose. Accordingly, the Na^+^ and K^+^ concentrations in HBSS were 141.4 mM and 5.8 mM, respectively. BS contained 140 mM NaCl, 1 mM CaCl_2_, 1 mM MgCl_2_, 5 mM glucose and 10 mM HEPES (pH 7.4). The basal K^+^ concentrations in BS were 4.0 mM or 5.4 mM. When the K^+^ concentration was increased in BS to more than 10 mM, the NaCl concentration was reduced to maintain the sum of KCl and NaCl concentrations at 150 mM.

Pipette and bath solutions were identical in patch clamp experiments and contained 120 mM NaCl, 2.8 mM KCl, 1 mM CaCl_2_, 2 mM MgCl_2_, 10 mM glucose and 10 mM HEPES (pH 7.2).

### Single Cell Imaging

The FLIPR membrane potential (FMP) dye is provided in a proprietary formulation containing additionally a membrane impermeant quencher (FLIPR Membrane Potential Assay kit BLUE, R8042, Molecular Devices, Biberach an der Riss, Germany). Since both dye and quencher have to be present at the same concentration in all solutions used in the experiments, we dissolved the content of one vial of the FMP formulation directly in 10 ml of recording solution. In our hands, dilutions with more than 15 ml solution per vial reduced the FMP fluorescence to less than a third (excitation: 520–540 nm; emission: 605 nm). The minimal loading time was determined in initial experiments by measuring FMP fluorescence intensities after exposing HEK cells, DRG neurons and feeder layer cells to FMP-containing HBSS at room temperature. The FMP fluorescence increased very rapidly in all these cells and constant intensities were measurable already 1–2 min after exposure to FMP. In all subsequent experiments, images were taken 10–15 min following loading with FMP at room temperature. Cell loading was performed in the recording solution, i. e., either HBSS or BS with basal K^+^.

**Figure 1:**
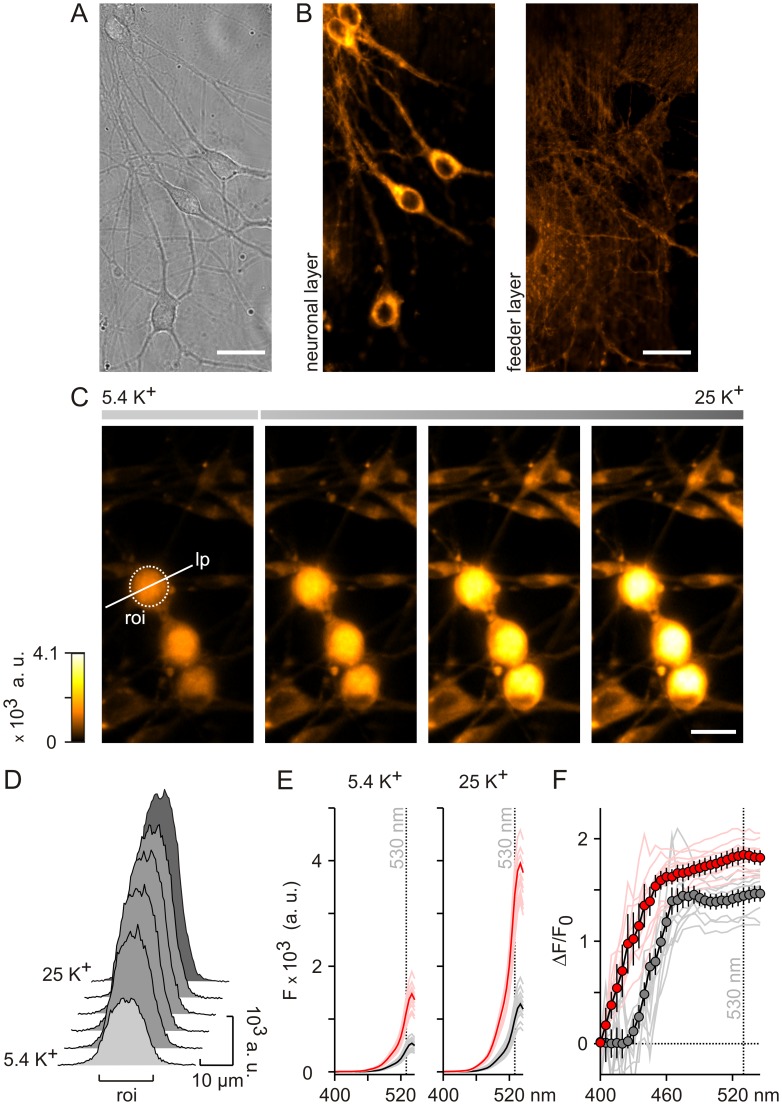
Intracellular localization and spectral properties of the FMP dye in neurons. (**A**) Phase contrast image of hippocampal neurons on top of astrocytic feeder layer. Scale bars, 20 μm. (**B**) Confocal-like optical sections of the neurons shown in (A) 15 min after labelling with FMP in Hanks' balanced salt solution (HBSS). Strong fluorescence of neurons (left panel) and low background of astrocytic feeder layer (right panel) show preferential labelling of neurons with intracellular localization of the FMP dye. (**C**) Epifluorescence images showing increase in FMP emission intensity upon depolarizing dorsal root ganglion (DRG) neurons by increasing KCl from a resting concentration of 5.4 mM (5.4 K^+^) to 25 mM (25 K^+^) in the basic solution (BS). Images of resting DRGs and intermediate images as fluorescence intensity increases are shown. roi, region of interest; lp, line profile; a. u., arbitrary units. (**D**) Cross-section line profiles of FMP fluorescence in a DRG neuron as indicated in (C). The example illustrates the homogenous increase in cytosolic fluorescence during transition of neurones from resting (5.4 K^+^). (**E**) Excitation spectra of the FMP dye in DRG neurons exposed to 5.4 K^+^ (left) and 25 K^+^ (right). Average spectra of neurons (bold red, n = 17) and astrocytic feeder cells (bold black, n = 36) are shown with examples of individual cells (light red and grey) for excitation wavelengths between 400 and 545 nm. (**F**) Relative changes of FMP fluorescence intensity calculated as ΔF/F_0_ from the spectra shown in (E). Average values of neurons (red circles) and astrocytic feeder cells (grey circles) are plotted with individual cell traces (light red and grey lines). Data is presented as mean ± SEM. The relative changes of the FMP fluorescence measured at 530 nm excitation displayed a high signal-to-noise ratio (F) independently of individual cell fluorescence intensities (E).

Initial FMP imaging of cells was performed on a structured illumination system (ApoTome, Carl Zeiss, Göttingen, Germany) in order to produce confocal-like sections for elucidation of labelling pattern. Excitation was centred at 534 nm and emission was detected at 605 nm using a customized filter set (exciter 534/20 nm, dichroic mirror 565 DCXR, emitter 605/70 nm, AHF analysentechnik, Tübingen, Germany). Further FMP imaging was performed using an iMIC-based imaging system (TILL Photonics, Gräfelfing, Germany) equipped with a Polychrome V that allows the software-controlled adjustment of excitation wavelengths (bandwidth 10 nm). The epifluorescence was detected at 605 nm using a customized FMP filter set (dichroic mirror 565 DCXR, emitter 605/70 nm, AHF analysentechnik) and images were captured with a 14-bit camera. FMP spectra were obtained by exciting the dye with increasing wavelengths between 400 nm and 545 nm in 5 nm steps. Since FMP produces a bright fluorescence, we chose the shortest possible exposure time of 5 ms. Under these conditions, the least intense FMP signals at 530 nm excitation displayed on average 100 counts/pixel above background. All measurements of the FMP fluorescence within regions of interest (roi) and line profiles (lp) were performed on background-subtracted images. Relative changes in the FMP fluorescence intensity (ΔF/F_0_) were calculated as the difference between fluorescence intensities within the roi (ΔF) divided by the initial intensity value (F_0_). For instance, in Fig. 1, the change in fluorescence intensity values between 25 mM and 5.4 mM K^+^ (ΔF) with respect to roi intensities in 5.4 mM K^+^ (F_0_).

Based on the spectral analysis (Fig. 1E–F), we selected the excitation wavelength of 530 nm and the exposure time of 5 ms for further experiments. Time courses were generated by taking images every 2 s. In general, it was possible to increase the sampling rate up to 20 frames/s without any signs of bleaching. ΔF/F_0_ was calculated as the change in roi intensities (ΔF) over time with respect to the average roi intensities of the first 10 images (F_0_).

The ratiometric dye SBFI [2] was used in conjunction with FMP to image internal Na^+^ concentrations and membrane potential in the same cells. SBFI-AM (Molecular Probes, Darmstadt, Germany) was dissolved in DMSO with 20% Pluronic F-127 and added to cells in HBSS at a final concentration of 5 µM SBFI-AM. We loaded the cells with SBFI-AM at 37°C for 45 min. Subsequently, SBFI-AM was washed and cells were incubated at room temperature for a further 15 min in HBSS containing FMP. Taking advantage of the filter change capability of the iMIC microscope, FMP and SFBI filter sets were exchanged automatically to obtain images of both FMP and SBFI fluorescence in co-labelled cells. We first took the FMP image with 530 nm excitation (exposure time 5 ms) using the FMP filter set (dichroic mirror 565 DCXR, emitter 605/70 nm, AHF analysentechnik). Afterwards, the SBFI filter set (dichroic mirror DCLP410, emitter LP470, TILL Photonics) was placed in position and SBFI images were obtained with 340 and 380 nm excitation (exposure time 10 ms). This cycle was repeated every 2 s to generate parallel time courses of the FMP and SBFI fluorescence in the same cells. SBFI ratio images were calculated on background subtracted images and data is presented as ratios F_340_/F_380_, where F_340_ and F_380_ represent fluorescence intensities at 340 and 380 nm, respectively.

### Photometry and Voltage Clamp

In order to determine changes in FMP fluorescence under defined membrane potentials, the photometric technique was used in combination with the voltage-clamp mode of the perforated patch-clamp technique. The voltage-clamp mode was preferred because it allows membrane potential changes to be achieved within microseconds, whereas those induced by current injection in current-clamp mode are much slower due to the membrane time constant of HEK cells at about 35 ms. As in imaging experiments, FMP was continuously present in the bath solution. The pipette solution contained 300 µM nystatin (Sigma-Aldrich, Hamburg, Germany) and no FMP. The membrane potential was clamped at desired values using an EPC-9 amplifier (HEKA Elektronik, Lambrecht/Pfalz, Germany) and the FMP fluorescence was measured using a photometry system (TILL Photonics), that was incorporated into the patch-clamp system. Basically, the FMP dye was excited with a Polychrome IV and the fluorescence of the voltage-clamped cell was detected at 605 nm with a photodiode-based fluorescence detector and a FMP filter (dichroic mirror 565 DCXR, emitter 605/70 nm, AHF analysentechnik). To measure the FMP fluorescence under voltage clamp conditions, the FMP dye was excited at a rate of 4 Hz with a 530 nm wavelength (exposure time 6 ms). At the same time, the membrane potential was changed with a series of voltage-clamp pulses from −60 mV to values between −80 and 40 mV in 20 mV increments. FMP measurements were started when the series resistance decreased below 20 MΩ. ΔF/F_0_ was calculated with respect to the FMP intensity within the first 1 s of recording at −60 mV.

### Compounds and Cell Stimulation

Ionotropic and metabotropic receptors were activated with L-glutamic acid, acetylcholine chloride, GABA, diazepam, (S)-3,5-DHPG, (S)-AMPA and NMDA (Tocris biosciences, Wiesbaden-Nordenstadt, Germany). (E)-Capsaicin (Tocris biosciences) and (-)-menthol (Sigma-Aldrich) were used to activate temperature sensors. In some experiments, the ionophores SQI-Pr (Calbiochem, Darmstadt, Germany) and valinomycin (Sigma-Aldrich) were used. Stock solutions were prepared by dissolving the compounds in either DMSO or distilled water and, just before stimulation, the compounds were further diluted to a 2X concentration in the recording solution, i.e., 2 times greater than the desired final concentration. Routinely, stimulation of cells was achieved by adding 2X solutions to the bath at a ratio of 1∶1 to avoid problems arising from slow mixing. The final DMSO concentration in bath was 0.001–0.005% v/v. In order to induce cell membrane depolarisation, the external K^+^ concentration was increased by adding recording solutions with the appropriated KCl concentration to the bath at a ratio 1∶1. All solutions used in the experiments contained FMP (10 ml solution per FMP vial).

## Results

### FMP Fluorescence in Neurons

Since primary cultures of neurons usually contain astrocytic feeder layers, we first compared the FMP-staining of hippocampal neurons and feeder cells in confocal-like optical sections (Fig. 1A–B). Dishes of mixed cell types were incubated for 15 min at room temperature in FMP-containing Hanks’ balanced salt solution (HBSS) with 5.4 mM KCl. Although both neurons and feeder cells exhibited FMP fluorescence, labelling was stronger in the neuronal layer (Fig. 1B). Furthermore, the highest FMP-fluorescence was primarily detected in neuronal somas. Labelling of the cell membrane or intracellular organelles was not observed at this optical resolution level, indicating that FMP accumulates preferentially in the cytosol. Next, we quantified the cytosolic distribution and intensity of the fluorescence emitted by the FMP dye in neurons and astrocytic feeder cells using an epifluorescence imaging system (Fig. 1C–E). As with hippocampal neurons (Fig. 1A–B), dishes with primary cultures of dorsal root ganglion (DRG) neurons were incubated with a basic solution (BS) containing the FMP dye in 5.4 mM KCl. By selecting cross-section line profiles (lp) through the centre of DRG cell somas, we determined the distribution of the FMP fluorescence whilst the extracellular KCl concentration was increased from a resting value of 5.4 mM to 25 mM in order to depolarise the cell (Fig. 1C). As depicted in Fig. 1D, the cross-section profiles indicated that the FMP fluorescence increased homogenously within neuronal somas until it reached eventually a higher intensity level upon raising the KCl concentration. Accordingly, we selected regions of interest (roi) containing the soma in order to measure the mean cell FMP fluorescence intensity in further analyses (Fig. 1C).

In order to determine the optimal wavelengths for monitoring FMP signals in neurons, the spectral properties of the FMP dye were investigated by exciting the dye with increasing wavelengths from 400 to 545 nm in the presence of 5.4 and 25 mM extracellular K^+^ (Fig. 1E). Essentially no FMP fluorescence was detected below 460 nm, while the fluorescence intensity increased progressively between 460 nm and 540 nm. At excitation wavelengths higher than 540 nm, we observed a decay of the FMP fluorescence intensity that might reflect the decay of light reflection of the dichroic mirror (see Methods). Both at basal and elevated K^+^, the FMP fluorescence intensities detected with excitation wavelengths above 460 nm were in DRG neurons 2.5–3.5 times higher than in feeder cells (Fig. 1E). Furthermore, the rise in extracellular K^+^ enhanced the FMP fluorescence intensity both in neurons and feeder cells, as expected for a KCl-induced depolarisation of the cell membrane. At 530 nm, the increase of the FMP fluorescence intensity induced by raising extracellular K^+^ was 2.5–3.3 fold both in neurons and feeder cells (Fig. 1E). Since FMP apparently accumulates in the cytosol (Fig. 1A–B), the differences in FMP signals between neurons and feeder cells shown in Fig. 1E might arise from the different morphology of the cells. In order to control for such effects, we calculated the relative changes of the FMP fluorescence intensity (ΔF/F_0_) with respect to the fluorescence intensity in 5.4 mM K^+^ (F_0_). Accordingly, ΔF represents the difference between fluorescence intensities in 25 mM and 5.4 mM K^+^. The spectral data converted into ΔF/F_0_ is shown in Fig. 1F. Since the FMP fluorescence intensities were negligible below 460 nm (Fig. 1E), the ΔF/F_0_ values were not reliable in the range of 400–460 nm. By contrast, ΔF/F_0_ was nearly independent from the excitation wavelength above 460 nm (Fig. 1F). In principle, any wavelength above 460 nm can therefore be used to excite the FMP dye. Since the dichroic mirror of the filter set selected for the present experiments (see Methods) efficiently reflects light up to 540 nm, an excitation wavelength of 530 nm was chosen as the basis for further experiments. At 530 nm, the ΔF/F_0_ induced by raising the external K^+^ concentration from 5.4 to 25 mM was on average 1.84±0.09 and 1.44±0.07 in DRG neurons and feeder cells, respectively.

### Membrane Potential-dependence of FMP Signals

**Figure 2:**
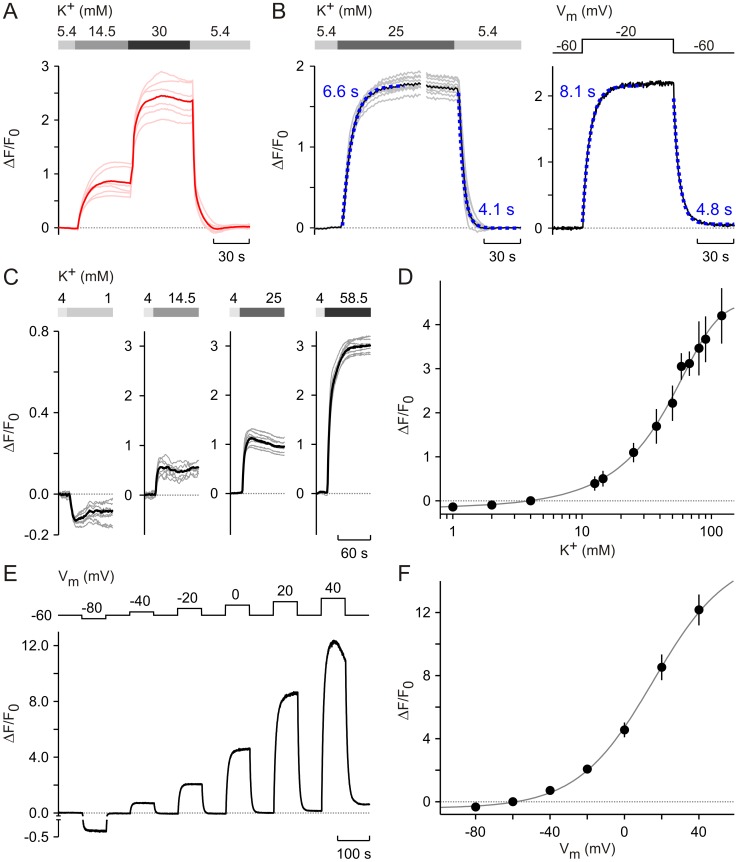
FMP dye responses to changes in cell membrane potential. (**A**) Reversibility of the FMP signal (ΔF/F_0_) assayed in DRG neurons as the cell membrane was depolarised and repolarised by changing the K^+^ concentration in BS. Bold red line represents mean response, n = 16; individual cell responses are shown as light red lines. (**B**) Time courses of FMP signals during high K^+^ pulses (left panel) and voltage-clamp pulses (right panel). HEK cells were exposed to the indicated K^+^ concentrations (left panel); mean (black) and single cell examples (grey) are shown. The membrane potential of HEK cells was clamped to the indicated potentials (right panel) using the perforated patch clamp technique and the FMP fluorescence was measured using a photometry system. The exponential fittings (blue) show that similar time constants were achieved for the rise and decay of FMP signals, when HEK cells were depolarised and repolarised using high K^+^ and voltage-clamp pulses. (**C**) FMP signals observed in HEK cells exposed to increasing K^+^ concentrations in BS. Mean (black) and single cell (grey) signals from example experiments are shown. K^+^ step protocols are depicted above graphs. (**D**) Relationship between ΔF/F_0_ and the KCl concentration in BS from experiments performed with HEK cells as illustrated in (C), n: 21–30. The reference K^+^ concentration was 4 mM and the data was fitted with an empirical sigmoidal function (grey line). (**E**) FMP signals elicited in HEK cells by voltage-clamp pulses. Photometric measurements and voltage clamp were performed as in (B). The voltage-clamp pulse protocol is shown above the FMP signal. (**F**) Relationship between ΔF/F_0_ and membrane potential (V_m_) obtained in combined voltage clamp and photometry experiments. The resting membrane potential was −60 mV. The grey line is a data fit with an empirical sigmoidal function; n = 5. Data is presented as mean ± SEM.

After choosing the optimal spectral conditions for assessing FMP (Fig. 1), we investigated the ability of the dye to monitor changes in membrane potential. Firstly, we examined whether FMP detects membrane potential changes induced by both raising and lowering the external K^+^ concentration. The FMP fluorescence was monitored in DRG neurons and stepwise KCl increases were made from 5.4 mM up to 30 mM, followed by a return to 5.4 mM (Fig. 2A). As illustrated in Fig. 2A, ΔF/F_0_ rose up quickly to a new plateau level with each increase of the extracellular KCl concentration and returned back to baseline levels upon lowering KCl to the starting concentration. Thus, FMP could monitor both depolarisation and repolarisation events in a reversible manner.

Next, the time taken for FMP signals to reach steady-state levels was determined during changes of the external KCl concentration and during the corresponding membrane potential changes (Fig. 2B). Human embryonic kidney (HEK) cells were incubated in FMP-containing BS and afterwards imaged whilst the external KCl concentration was raised and lowered between 5.4 mM and 25 mM (Fig. 2B, left panel). In order to estimate the membrane potential changes (ΔE) induced by manipulating external K^+^, we used expressions that were derived from both the Nernst and Goldman-Hodgkin-Katz (GHK) equations [19] under the assumption that the internal ion concentrations remain unchanged during the short exposures to elevated KCl. Based on the Nernst equation [20], the ΔE induced by manipulating the external K^+^ concentration can be calculated as
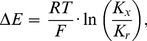
(1)where K_x_ and K_r_ represent the changing and reference external K^+^ concentrations, respectively. RT/F = 25.43 mV at 22°C. Accordingly, raising and lowering external K^+^ between 5.4 mM and 25 mM were expected to generate a ΔE of 38.97 mV. Since the Na^+^ concentration was reduced to maintain a constant osmolarity (see Methods), we also used an alternative approach based on the GHK equation [19]. Accordingly, the ΔE induced by manipulating both external K^+^ and Na^+^ concentrations can be calculated as
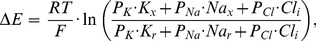
(2)where PK, PNa and PCl represent the permeability coefficients of the cell membrane for the individual ions. Nax and Nar represent the changing and reference external Na+ concentrations, respectively, and Cli denotes the internal Cl− concentration. Considering that the permeability coefficients of HEK cells are not known, we estimated ΔE using two models that differ basically in the contribution of PNa to the resting potential in HEK cells (see Fig. S1). Assuming a high PNa (PK : PNa :PCl = 1.00∶ 0.18∶ 0.10), the ΔE induced by changing external K+ between 5.4 mM and 25 mM amounts to 10.36 mV whilst a low PNa (PK : PNa :PCl = 1.00∶ 0.10∶ 0.10) gives a ΔE of 15.06 mV. Thus, the changes of external K+ indicated in experiment Fig. 2B (left panel) were expected to produce depolarisations with a maximum value of +40 mV. In order to relate ΔF/F0 to membrane potential changes, we used the voltage-clamp mode of the perforated patch-clamp technique combined with photometry and measured the FMP fluorescence under defined membrane potentials. Using this method, we imitated the ΔE expected to occur in the experiments shown in Fig. 2B (left panel) by depolarising and repolarising the HEK cells by 40 mV (Fig. 2B, right panel). Under both experimental conditions, the rise and decay of ΔF/F0 followed exponential time courses. The corresponding time constants were in the range of seconds and, notably, were very similar with both experimental approaches (Fig. 2B, left and right panel), suggesting that FMP does indeed report changes in membrane potential induced by manipulating the external K+ concentration. A good correlation between FMP responses and changes of membrane potential induced by high K+ was also reported for current-clamped CHO cells [11]. Since the photometric measurements in voltage-clamped cells represent the best experimental condition to study FMP fluorescence, our results indicate that the FMP dye resolves changes of the membrane potential with a time resolution of seconds (Fig. 2B, right panel).

An investigation of the membrane potential-dependence of ΔF/F_0_ was then performed in HEK cells with free-running membrane potential and in voltage-clamped HEK cells (Fig. 2C–F). Taking 4 mM as reference, the external K^+^ concentration was changed between 1 mM and 120 mM (Fig. 2C–D). Using eq. 1, it can be calculated that these changes of the K^+^ concentration might generate hyperpolarisations and depolarisations between −35.25 mV and +86.49 mV, whereas eq. 2 predicts membrane potential changes between −2.48 mM and +35.17 mV for high P_Na_. In order to cover the widest range, the membrane potential was varied from a resting potential of −60 mV to values between −80 mV and +40 mV in the voltage-clamp experiments (Fig. 2E–F). As can be predicted from the experiments shown in Fig. 2B, ΔF/F_0_ decreased below zero when external K^+^ was lowered below the reference level and when cells were hyperpolarised to −80 mV, and conversely, elevations of the external K^+^ concentration and depolarisations up to +40 mV enhanced ΔF/F_0_ (Fig. 2C, E). The relationship between ΔF/F_0_ and the external K^+^ concentration displayed a sigmoidal shape (Fig. 2D). Notably, a sigmoidal relationship was also observed between ΔF/F_0_ and V_m_ (Fig. 2F). Furthermore, a sigmoidal relationship was also seen between ΔF/F_0_ and the external K^+^ concentration in various neuronal cultures (see below), indicating that the sigmoidal relationship between ΔF/F_0_ and membrane potential is a general feature of FMP and is not cell-type-dependent.

**Figure 3:**
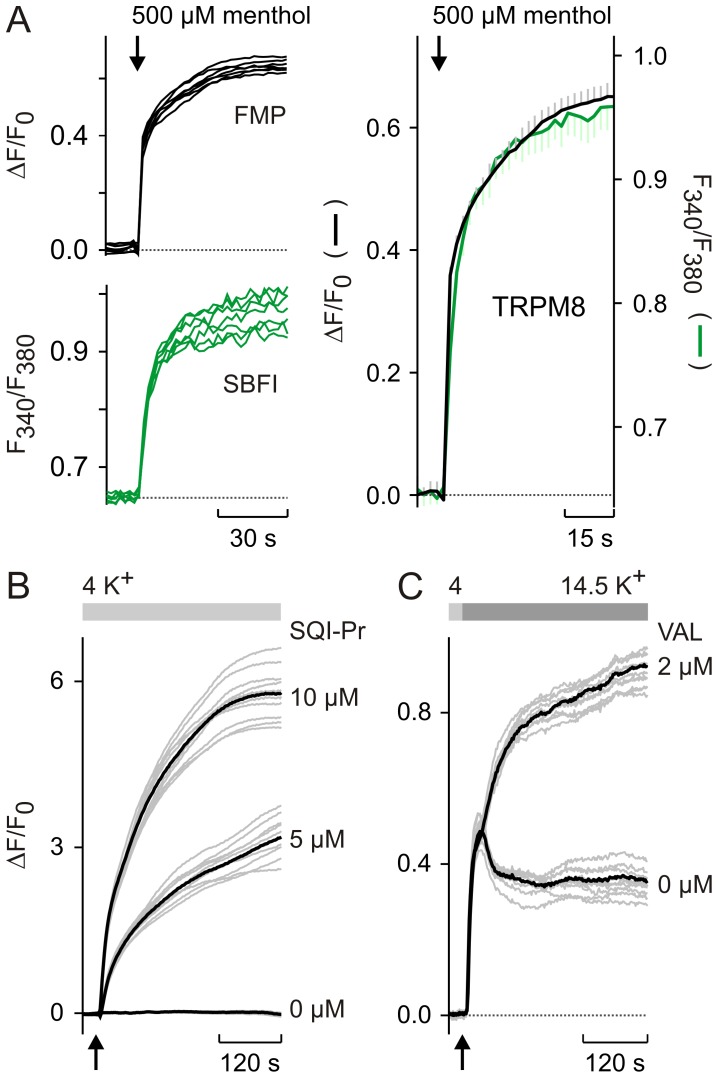
Ionic contribution to membrane potential changes detected by the FMP dye. (**A**) HEK cells over-expressing the Na^+^-selective channel TRPM8 were co-labelled with both FMP and SBFI, and treated with 500 µM menthol in HBSS. Cells were monitored for changes in membrane potential and intracellular Na^+^ using FMP and SBFI dyes, respectively (left panels). Shown are FMP (ΔF/F_0_) and SBFI (F_340_/F_380_) signals of individual cells. Upon overlaying average FMP (black) and SBFI (green) signals, similar responses are seen (right panel). (**B**) FMP signals induced by the Na^+^ ionophore SQI-Pr. Two different concentrations of SQI-Pr (5 µM and 10 µM) were tested. Mock applications (0 µM) were performed as controls. (**C**) FMP responses to high K^+^ steps in the absence (0 µM) and presence (2 µM) of the K^+^ ionophore valinomycin (VAL). Experiments in (B) and (C) were carried out with HEK cells in BS. Average traces are shown in bold and individual traces are in grey; arrows indicate the time point of mock, SQI-Pr and valinomycin applications.

The photometric measurements of the FMP fluorescence in voltage-clamped cells (Fig. 2E–F) provided direct evidence for the membrane potential dependence of FMP signals. Nonetheless, we wished to determine whether FMP can detect membrane potential changes irrespective of which ion types or ion channels give rise to it (Fig. 3). To achieve this, we first performed imaging experiments with HEK cells over-expressing the Na^+^ selective channel TRPM8 [13]. As previously described [21], [22], TRPM8 was activated with 500 µM menthol at room temperature. To correlate changes of membrane potential with the increase of the internal Na^+^ concentration, we simultaneously imaged the FMP fluorescence and the fluorescence of the ratiometric dye SBFI (see Methods). While control HEK cells were basically not responsive to menthol (not shown), both FMP and SBFI detected large responses to menthol treatment in TRPM8-expressing cells (Fig. 3A, left panel). Overlaying the traces showed that the SBFI fluorescence ratios and the relative changes of the FMP fluorescence had identical time courses (Fig. 3A, right panel), demonstrating that FMP can detect membrane potential changes arising from the opening of Na^+^ permeable channels. Supporting this suggestion, the treatment of HEK cells with the Na^+^ ionophore SQI-Pr resulted in dose-dependent increases of the FMP signal (Fig. 3B), as expected, since SQI-Pr creates an artificial Na^+^ conductance in the plasma membrane [23]. The electrogenic K^+^ ionophore valinomycin [24] enhanced the FMP signals produced by increasing external K^+^ from 4 mM to 14.5 mM (Fig. 3C). Taken together, the results shown in Fig. 3 demonstrate that changes in membrane potential arising from both Na^+^ and K^+^ redistribution could be detected by FMP.

### Quantitative Analysis of FMP Signals

Since FMP appears to move across the cell membrane according to changes in membrane potential, we used the Boltzmann distribution to develop a series of equations that relate the relative changes in the FMP fluorescence intensity (ΔF/F_0_) to changes of membrane potential (ΔE) and external K^+^ concentration (K_x_/K_r_). In the present study, ΔF/F_0_ was calculated as
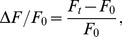
(3)where F_t_ and F_0_ represent fluorescence intensities at a given time point (t) and at the beginning (t = 0) of imaging, respectively. Thus, ΔF/F_0_ can be related to the absolute fluorescence change F_t_/F_0_ as follows



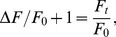
(4)Assuming that the fluorescence intensity is linearly proportional to the FMP concentration in the cytosol (FMP_i_), eq. 4 can be rewritten as
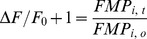
(5)


Since the external dye concentration (FMP_e_) likely remains constant during recordings due to the large volume of the recording chamber compared to the cell volume, eq. 5 can be rearranged to
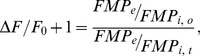
(6)where FMP_e_/FMP_i_ represents the partitioning of the dye across the cell membrane. If FMP is distributed as a charged molecule across the cell membrane in accordance with membrane potential, the Boltzmann equation predicts that
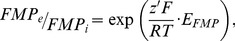
(7)where z′ and EFMP denote the apparent charge and equilibrium potential of FMP. R and F are the gas and Faraday’s constants and T represents the absolute temperature. Accordingly, eq. 6 can be rewritten to include expressions for the membrane potential:
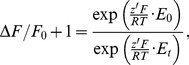
(8)where E0 and Et are the membrane potentials that determine the distribution of FMP across the membrane at the corresponding time points. Finally, rearranging eq. 8, we obtain the equation describing the relationship between ΔE and ΔF/F0,
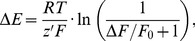
(9)in which, membrane potential changes (ΔE) are defined as
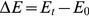
(10)and, the depolarisation and hyperpolarisation of the cell membrane correspond to ΔE >0 and ΔE <0, respectively. Similarly, eq. 9 states that positive and negative values of ΔF/F0 reflect depolarisation and hyperpolarisation events, respectively.

It is well known that small changes around the resting external K^+^ concentration do not necessarily induce depolarisation and hyperpolarisation events that follow the Nernst equation [25]. However, for high external K^+^ concentrations, less deviations from the Nernst equation are expected and eq. 1 can be used to estimate ΔE. Accordingly, by combining eq. 1 with eq. 9 and rearranging terms, we finally obtain the equation that describes the relationship between ΔF/F_0_ and the fraction K_x_/K_r_, which represents the change of the external K^+^ concentration (K_x_) with respect to a reference (K_r_) concentration:
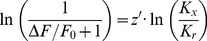
(11)


To take into account changes both in external K^+^ and Na^+^ concentrations, eq. 2 and eq. 9 can be combine to obtain
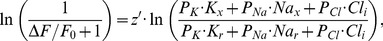
(12)which describes the relationship between ΔF/F_0_ and the changing external K^+^ and Na^+^ concentrations (K_x_, Na_x_) with respect to reference concentrations (K_r_, Na_r_). The permeability coefficients P_K_, P_Na_ and P_Cl_ determine the relative contribution of the individual ions to the changes in the membrane potential. The internal Cl^−^ concentration (Cl_i_) is assumed to be a constant.

**Figure 4:**
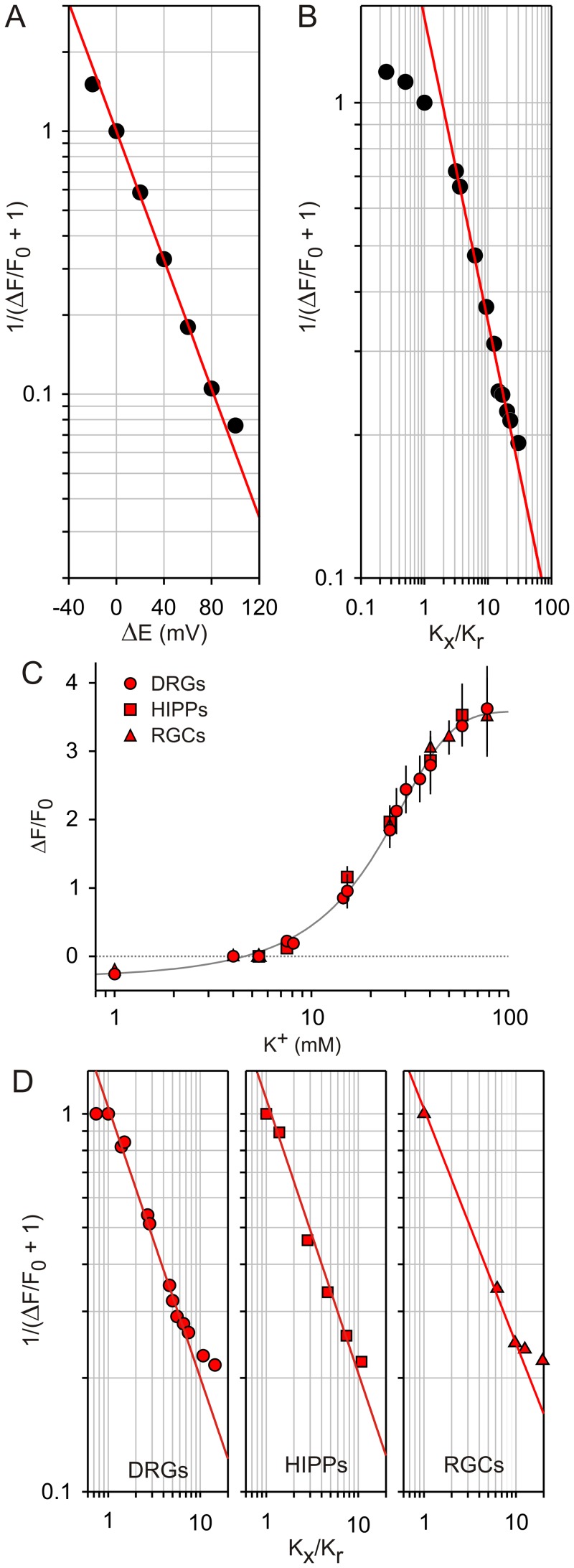
Relationship between FMP responses, membrane potential and the external K^+^ concentration. (**A**) Mean values of the relative changes of the FMP fluorescence (ΔF/F_0_) shown in Fig. 2F were transformed and fitted with eq. 9. For RT/F = 25.43 mV (at 22°C), the best fit to the data indicates that the apparent charge of FMP (z′) has a value of −0.71. Changes of membrane potential (ΔE) are given with respect to −60 mV. (**B**) Mean values of the data shown in Fig. 2D were transformed using the K^+^ concentration of 4 mM as reference (K_r_). The fit of the data with eq. 11 indicates that z′ = −0.64. (**C**) Graph showing relationship between the extracellular KCl concentration and ΔF/F_0_ for different neuronal cell types. ΔF/F_0_ was measured in experiments similar to those shown in Fig. 2C, in which high K^+^ pulses were applied from a reference K^+^ concentration of either 4 mM or 5.4 mM (solution BS). Hippocampal neurons (HIPPs), n = 17; DRGs, n = 12; RGCs, n = 15. A sigmoidal relationship between the KCl concentration and ΔF/F_0_ is apparent. (**D**) Mean values of the data shown in (C) were transformed and fitted with eq. 11, where the fraction K_x_/K_r_ represents the change of the external K^+^ concentration (K_x_) with respect to a reference (K_r_) concentration that was either 4 mM or 5.4 mM. Calculated z′ values are: −0.71 (DRGs); −0.72 (HIPPs); −0.62 (RGCs).

**Figure 5:**
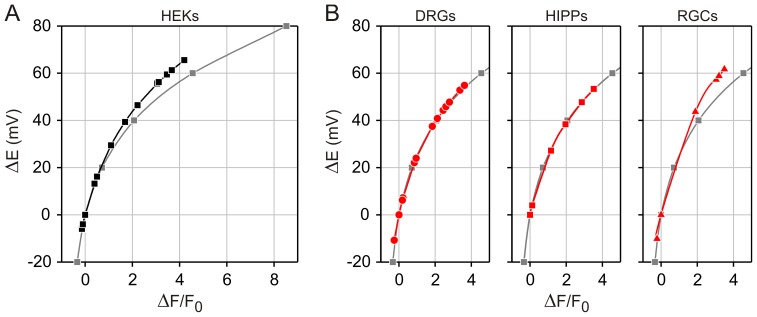
Calibration of FMP signals measured with a single cell imaging system. Changes of membrane potential (ΔE) induced by high K^+^ steps were calculated with eq. 9. RT/F = 25.43 mV (22°C). ΔF/F_0_ values were from Fig. 2C–D and Fig. 4C. (**A**) FMP signals measured in HEK (black). z′ = −0.64. (**B**) FMP signals from the indicated neuronal cell types (red). z′: −0.71 (DRGs); −0.72 (HIPPs); −0.62 (RGCs). For comparison, the plot ΔE vs. ΔF/F_0_ (grey) obtained using a photometric system on voltage-clamped HEK cells (Fig. 2 F) is superimposed on the graphs.

The relationships of ΔF/F_0_ to the membrane potential and to external K^+^ concentration were not linear and accounted best with empirical sigmoidal functions (Fig. 2D–F). Using logarithmic transformations of the voltage-clamp data shown in Fig. 2F, it can be estimated that a *e*-fold (2.72-fold) increase of the FMP fluorescence, which corresponds to a ΔF/F_0_ value of 0.72, is achieved with a 40 mV depolarisation. In order to allow more quantitative analysis, however, we expressed the data as 1/(ΔF/F_0_+1) which is the main term in eq. 9, 11 and 12. Similarly, we preferred to use ΔE and K_x_/K_r_ for further calculations instead of absolute membrane potentials and external K^+^ concentrations. As shown in Fig. 4, the apparent non-linearity of the raw data is largely overcome with this transformation of the data. Since the membrane potential is best controlled in voltage-clamped cells, we first fitted eq. 9 to the transformed data and found a perfect overlap of the theoretical curve to the data (Fig. 4A). Furthermore, the apparent charge of FMP (z′) that best fits the data was −0.71. Since FMP is a proprietary formulation, it is not possible to correlate z′ with the chemical structure of the dye. Thus, we preferred to interpret z′ values as empirical indicators of the voltage sensitivity of the dye because z′ determines simply the slope in eq. 9. In this interpretation, for instance, the voltage sensitivity of a diffusible dye is inversely proportional to z′. Analyses of the voltage sensitivity of other dyes have not been done in voltage clamped cells, but it has been reported that VSDs can have anionic or cationic structures [5] and, thus, the negative z′ value of FMP is not unexpected. An alternative approach is to determine z′ using data from experiments with high K^+^ steps and eq. 11 and 12. For K^+^ concentrations above 10 mM, fittings of eq. 11 to the data revealed a z′ value of −0.64 (Fig. 4B). Similarly, we found z′ values between −0.72 and −0.62 in neuronal cultures that were challenged with high K^+^ steps (Fig. 4D). Thus, the z′ values obtained with eq. 11 largely resemble those obtained with eq. 9 in voltage-clamped cells (Fig. 4A and B). In contrast, fittings of eq. 12 to the data generated z′ values that strongly differed from the value obtained in voltage-clamped cells (Fig. S1A). We found that z′ was −1.48 and −1.04 for high P_Na_ (P_K_ : P_Na_ : P_Cl_ = 1.00∶ 0.18∶ 0.10) and low P_Na_ (P_K_ : P_Na_ : P_Cl_ = 1.00∶ 0.10∶ 0.10), respectively. In order to determine the accuracy of the z′ values obtained with high K^+^ steps, we used the ΔF/F_0_ measured photometrically under voltage clamp, i.e., under known membrane potentials (Fig. 2F) to calculate ΔE using eq. 9 and compared these predicted values with the ΔE of the voltage-clamp steps (Fig. S1B). This comparison demonstrated a close correlation between the applied ΔE and the values predicted using the z′ obtained with eq. 11 (Fig. 4B) whilst the ΔE predicted with z′ values obtained with eq. 12 were up to one-half of the ΔE applied in the voltage clamp experiments. For instance using the z′ value of −0.64 obtained with high K^+^ steps, it can be calculated with eq. 9 that the plateau of ΔF/F_0_ in the voltage-clamp experiment shown in Fig. 2B corresponds to a ΔE of +43.65 mV, which is slightly higher than the 40 mV depolarisation applied in this particular example. In contrast, ΔE is far below +40 mV with the z′ values of −1.48 and −1.04 (+19.30 mV and +27.43 mV). Finally, we used the z′ values measured with high K^+^ steps in HEK cells (Fig. 2D, 4B) and in neuronal cell cultures (Fig. 4C–D) to calibrate FMP signals. As shown in Fig. 5A, the hyperpolarisations and depolarisation induced with high K^+^ steps in HEK cells (Fig. 2C–D) ranged between −5.95 mV and +65.56 mV. The relationship between ΔE and ΔF/F_0_ is approximately linear with an average ΔF/F_0_ of 0.5 per 10 mV depolarisation within the physiological range of −10 mV to +50 mV. Furthermore, the calibrated imaging signals correlated well with the signals measured photometrically in voltage-clamped cell both in HEK cells (Fig. 5A) and neuronal cell cultures (Fig. 5B). Thus, it appears that the z′ values measured with high K^+^ steps and calculated with eq. 11 agree well with the z′ of voltage-clamped cells (Fig. 4A–B) and are suitable for calibration of FMP signals obtained in single cell imaging systems.

### Imaging Neuronal Activity with FMP

**Figure 6:**
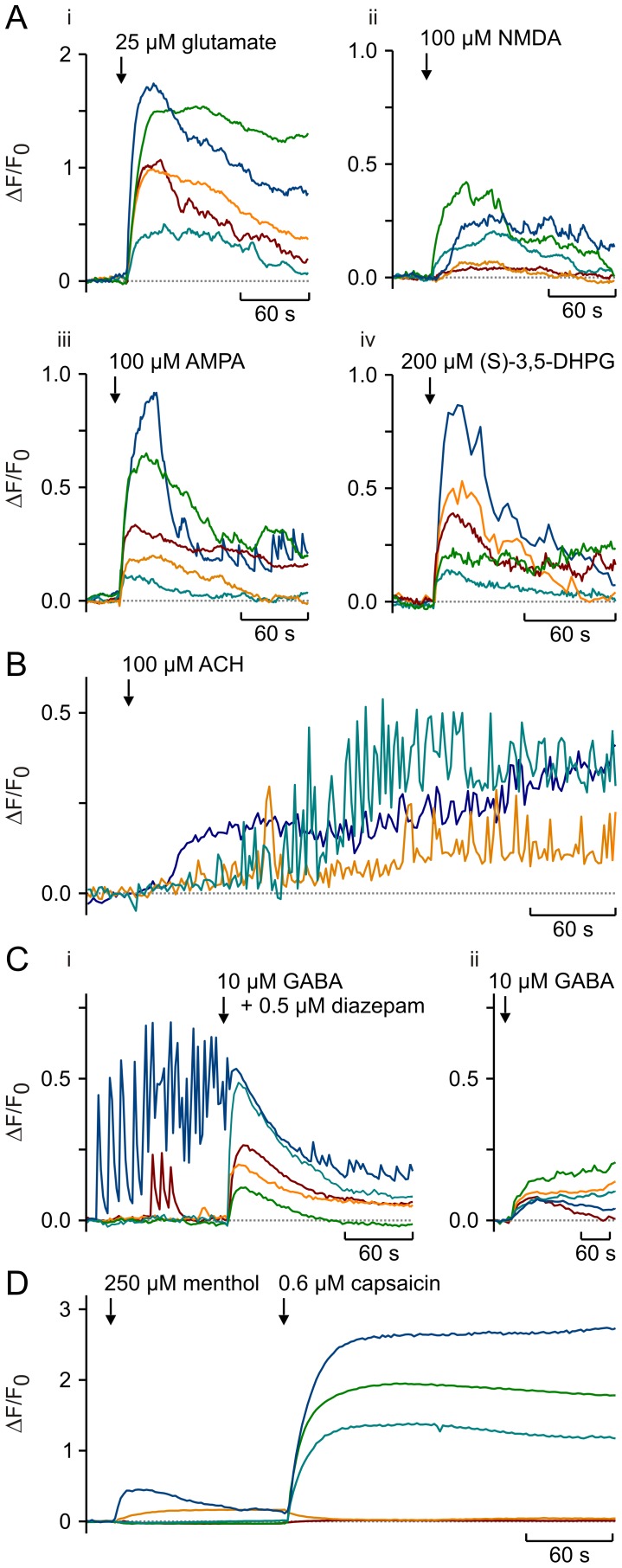
FMP responses during stimulation of neurons with various neurotransmitters. (**A**) FMP signals elicited by activation of ionotropic and metabotropic glutamate receptors. Retinal ganglion cell (RGCs) were treated with 25 µM glutamate (i), 100 µM NMDA (ii) and 100 µM AMPA (iii). Septal neurons were exposed to 200 µM (S)-3,5-DHPG (iv). (**B**) Cortical neurons were treated with 100 µM acetylcholine (ACH) resulting in rapid oscillations of the FMP fluorescence. (**C**) The spontaneous oscillations of the FMP fluorescence observed in some hippocampal neurons were silenced upon addition of 10 µM GABA and 0.5 µM diazepam (i). Addition of 10 µM GABA in the presence (i) and absence (ii) of diazepam slightly enhanced the FMP fluorescence in most hippocampal neurons. (**D**) Dorsal root ganglion neurons (DRGs) were treated with 250 µM (-)-menthol to activate cold receptors, followed by 0.6 µM (E)-capsaicin to activate heat receptors. Experiments were performed in HBSS. Shown are representative FMP signals of individual neurons depicted in colour.

In order for FMP to be useful for studying physiological changes in neuronal membrane potential, it would need to be able to detect changes induced by the action of neurotransmitters. To determine the effect of an array of neurotransmitters, different neuronal cell types were cultured. Firstly, the classic excitatory neurotransmitter glutamate was addressed using retinal ganglion cells and septal neurons that express various types of ionotropic and metabotropic glutamate receptors [26], [27]. Both glutamate and agonists of glutamate receptors such as NMDA, AMPA and (S)-DHPG elicited clear increases of FMP fluorescence in the recipient cells (Fig. 6A). Next, acetylcholine was used to treat cortical neurons [28]. Following a short delay, acetylcholine induced spike-like FMP signals likely reflecting action potential firing (Fig. 6B). By contrast, treatment of spontaneously active hippocampal neurons with GABA combined with diazepam [29] caused a cessation of spontaneous active firing, demonstrating that the effects of inhibitory neurotransmitters can also be detected using FMP (Fig. 6C). However, GABA treatment of non-active hippocampal neurons caused an increase of the FMP fluorescence rather than a decrease as expected for a hyperpolarisation. This reflects the well-known depolarizing action of GABA on immature neurons, which has been shown to arise from Cl^−^ effluxes supported by the high internal Cl^−^ concentrations present in these neurons [30)]. Finally, DRGs were treated with menthol and (E)-capsaicin to activate cold and heat receptors encoded by transient receptor potential (TRP) channel proteins [31]. As expected for the heterogeneous expression of thermosensitive TRP channels in DRGs [32], we observed responses to either menthol or (E)-capsaicin as well as to both compounds in the same DRGs (Fig. 6D).

**Figure 7:**
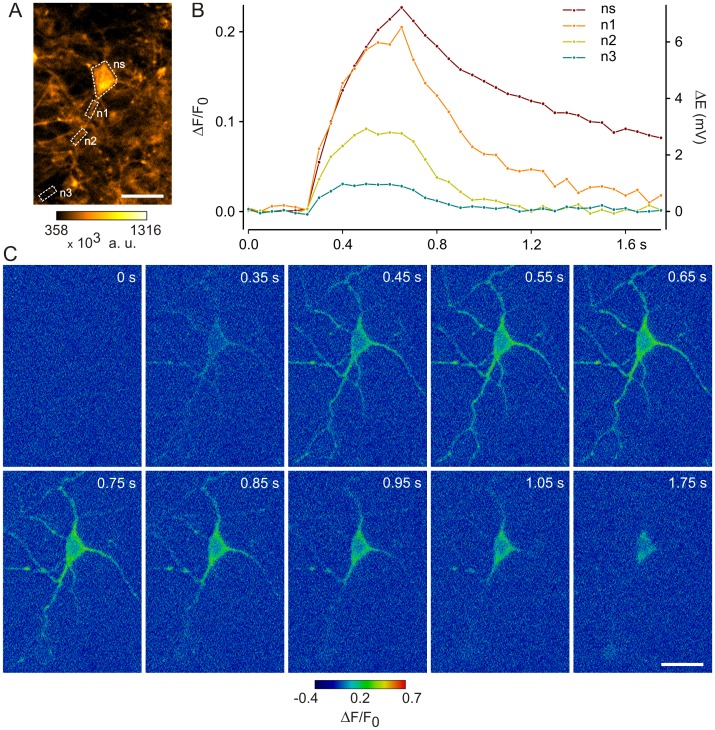
Monitoring of spontaneous neuronal activity with FMP. (**A**) FMP fluorescence in hippocampal neurons. The image shows the basal FMP fluorescence (530 nm excitation; 605 nm emission). Regions of interest on the neuronal soma (ns) and along neurites (n1–n3) are marked. Scale bars, 20 µm. (**B**) Time courses of ΔF/F_0_ recorded on the neuronal soma and neurites as indicated in A. The signals were calibrated with eq. 9. z′ = −0.72. RT/F = 25.43 mV (22°C). Images were sampled at a rate of 20 frames/s. (**C**) Single frames of neuronal activity recorded at the indicated time points. Images of relative FMP fluorescence (ΔF/F_0_) were computed pixel-by-pixel.

Since the recordings of FMP fluorescence shown so far were carried out with regions of interest placed on the cell soma, we finally recorded FMP signals in the neurites of hippocampal cells that were cultured at high density for 10 days (Fig. 7A). FMP imaging was performed with the highest sampling speed available in our imaging system, which is 20 Hz (20 frames/s). As shown in Fig. 7B–C, the FMP fluorescence increased spontaneously both in the soma and neurites. Typically, ΔF/F_0_ rose quickly and decayed slower. Using the calibration procedure shown in Fig. 5, such changes of ΔF/F_0_ correspond to less than 10 mV (Fig. 7B). Similarly, the neuronal responses to neurotransmitters shown in Fig. 6 correspond to 2.5–50 mV. Collectively, the data presented in Fig. 6 and 7 demonstrate that FMP can detect the action of multiple neurotransmitters on a whole array of neuronal cell types as well as spontaneous neuronal activity.

## Discussion

In the present study, we have examined the FMP dye as a tool to image membrane potential in neurons using epifluorescence-based systems that are commonly employed in single cell imaging. The subcellular localisation and the spectral properties of FMP were determined by analysing confocal-like images and excitation spectra. Direct information on the relationship between membrane potential and FMP fluorescence changes was obtained in experiments that combined voltage clamp and photometric techniques. On the basis of this data, we developed a theoretical background that supports the quantitative application of FMP in membrane potential imaging.

Since the germinal experiments with optical techniques in neurons [33], several VSDs have been synthesized and used to image changes of membrane potentials in vivo and in vitro in a variety of preparations [5], [6]. Underlying the voltage sensing of synthetic chromophores, one major mechanism is the redistribution and partitioning of the dye across the cell membrane according to the electric field [7]. In this instance, the dye moves into or out of the cell changing its concentration and hence the fluorescence intensity within the cell. By contrast, changes of the electronic structure of the chromophores underlie the voltage sensitivity of some membrane-bound VSDs [7]. Using confocal-like optical sections of various cell types labelled with FMP, we first determined the subcellular localisation of the FMP fluorescence and found no indication for a preferential labelling of cell membranes (Fig. 1A–B, 7C). Moreover, the FMP fluorescence was evenly distributed within the cytosol, suggesting that FMP belongs to the group of VSDs that move into and out of the cells following changes of membrane potential. This suggestion was further supported by cross-section analysis of neurons depolarised with high external K^+^, in which the FMP fluorescence increased within the cytosol as a consequence of membrane depolarisation (Fig. 1C–D). Furthermore, our characterisation of the FMP dye using an epifluorescence imaging system showed that the dye reports on depolarisation and repolarisation events as well as on hyperpolarisation of the membrane (Fig. 2). As for most VSDs [7], other issues concerned with the applicability in neuronal imaging are the fluorescence quantum yield and the signal-to-noise ratio, which determine the amplitude of the signals and the single neuron resolution as well. FMP was originally developed for plate readers [10] and, accordingly, high fluorescence intensities were expected. Using an epifluorescence system optimised for single cell imaging, we indeed obtained robust FMP signals with the shortest possible exposure time of 5 ms independently of the cell type. Under these conditions, the maximal fluorescence intensities were approximately 3 times higher in neurons than in feeder layer cells (Fig. 1E), making the neuronal FMP fluorescence easily detectable over background. Likely, the different three-dimensional structure of cultured neurons and feeder cells might explain the higher fluorescence levels in neurons growing on co-culture plates. Additionally, we have tested a series of filter sets that might be used to image the FMP fluorescence and found that the emitted fluorescence can be best detected with a filter centred on 605 nm [34]. By running excitation spectra, we found that although the FMP dye can be excited with wavelengths between 460 and 540 nm (Fig. 1E), the most stable measurements of the relative fluorescence intensity ΔF/F_0_ were obtained with the excitation wavelength of 530 nm (Fig. 1F), making it very easy to tailor filter sets for any single cell imaging system. These spectral properties make FMP an ideal candidate for co-labelling of cells with ion indicators. As a proof of principle, we have imaged simultaneously the internal Na^+^ concentration with SBFI and the membrane potential with FMP in cells expressing the cold sensor TRPM8 (Fig. 3A). As expected for a Na^+^-selective ion channel [31], we observed a near perfect correlation between the SBFI and FMP signals upon activation of TRPM8 with menthol, indicating that FMP is fast enough to detect changes of membrane potential induced by the opening of ion channels. Similarly, we have combined FMP and the Ca^2+^ indicator FURA-2 in previous studies of the Ca^2+^-dependent activation of TRPC5 ion channels [35]. Furthermore, the dye apparently does not interfere with responses to activators of glutamate, acetylcholine, GABA, menthol and capsaicin receptors in a variety of neuronal cultures (Fig. 6). We therefore conclude that FMP is perfectly suited to study the electrical activity in neuronal cultures with conventional single cell imaging systems.

As expected for a dye that likely diffuses across the plasma membrane, the increases and decreases in ΔF/F_0_ upon changes of the membrane potential were not instantaneous (Fig. 2 and 3). In order to determine the time constants of ΔF/F_0_ changes, we compared the time courses of ΔF/F_0_ during voltage-clamp steps and during stepwise increases of the external K^+^ concentration (Fig. 2B). Both the increase and decay of ΔF/F_0_ followed exponential time courses, which appeared independent of the depolarisation strength in voltage-clamped cells. The time constants of ΔF/F_0_ changes were in the range of 4–8 s, whereby increases were approximately 2 times faster than decreases of ΔF/F_0_ during voltage-clamp and high K^+^ steps. Although this time resolution is definitely too slow for the resolution of single action potentials, it allows recordings of the “slow” component of responses to ligands of membrane receptors (Fig. 6). Occasionally, we observed oscillations of the FMP fluorescence upon activation of membrane receptors (e.g., Fig. 6B). Similar oscillations were observed particularly in long-term hippocampal cultures (Fig. 7). Using the maximal sampling rate of our system (20 frames/s), we were able to resolve the time course of the oscillations both in the neuronal soma and in thin structures such as neurites (Fig. 7A–C), indicating that FMP is suitable to study electrical activity in the whole neuron. Since FMP cannot report single action potentials, the oscillations of the FMP fluorescence seen in neuronal cultures more likely reflect trains of action potentials. Using the calibration procedure shown in Fig. 5, the spontaneous activity and the responses to neurotransmitters correspond to depolarisations between 2.5 and 50 mV.

In comparative studies of VSDs in plate readers, it has been shown that the time resolution of FMP is 4 times faster in comparison with DiBAC_4_ [3] and that the signal amplitude is significantly greater [10]. However, it still does not compare to the temporal resolution possible with non-diffusion based dyes such as the ANEP dyes, which have been used for recording single action potentials [36]. It should be noted, though, that these dyes have very low voltage sensitivity. For example, di-8-ANEPPS has been reported to have relative fluorescence changes of only 2.5% per 100 mV change [37]. Ratiometric measurements improve the voltage sensitivity of di-8-ANEPPS to a 15% ratio change per 100 mV [38]. In general, the small signal-to-noise ratios are not optimal for accurate quantification of small membrane potential changes, supporting our choice of FMP for imaging membrane potential in neurons. In contrast, the ANNINE dyes have reported higher voltage sensitivities resulting in improved signal-to-noise ratios whilst maintaining fast response times in the sub-millisecond range [39]. ANNINE-6plus has, for instance, a voltage sensitivity of 10% per 100 mV [40]. However, laser light sources are needed to obtain sufficient excitation of ANNINE dyes [39]. Similarly, FRET-based VSDs have response times of 400 µs –500 ms and voltage sensitivities of 10–20% per 100 mV [41] but FRET measurements require equipment for dual-emission ratiometric imaging. Here, we report ΔF/F_0_ values for the FMP dye in the order of 12 for a 100 mV depolarisation and, on average, 0.5 per 10 mV for depolarisations below 50 mV (Fig. 5), indicating that the voltage sensitivity of FMP is at least 50% per 10 mV in the physiological range. This high voltage sensitivity combined with bright emission fluorescence (Fig. 1) and high spatial resolution (Fig. 7) make the FMP dye suitable to study neuronal activity with conventional single cell imaging systems.

Finally, the results of the present study suggest that in order to quantify FMP signals, one must first determine the z′ value for a specific preparation using high K^+^ steps and eq. 11. Subsequently, this z′ value can be inserted into eq. 9 to estimate the actual changes of membrane potential. Considering that voltage-clamp amplifiers are not usually available in most imaging systems, adjusting the membrane potential with the external K^+^ concentration appears to be the method of choice when quantitative analysis of the FMP fluorescence is needed. An alternative approach would be to use Na^+^ ionophores in combination with increases in the external Na^+^ concentration because FMP appears to report membrane potential changes independent of the ion type and ion channel type involved (Fig. 3). Since the ease of application and quantification compares to FURA-2, we believe that FMP imaging has the potential to become an established method for imaging membrane potential.

## Supporting Information

Figure S1 **Influence of membrane ion permeabilities on calculations of the apparent charge (z′) of the FMP dye.** (**A**) Determination of z′ considering the contribution of K^+^, Na^+^ and Cl^−^ ions to changes in the membrane potential. An approach based on the Goldman-Hodgkin-Katz equation was used to estimate z′. Accordingly, the data obtained with high K^+^ steps (Fig. 2D) was fitted with eq. 12. K_x_ and Na_x_ represent the changing external K^+^ and Na^+^ concentrations, respectively. In order to keep constant the osmolarity of high KCl solutions, Na_x_ was reduced to maintain K_x_+Na_x_ = 150 mM for K_x_>10 mM (see Methods). The reference concentrations K_r_ and Na_r_ were 4 mM and 140 mM, respectively. An internal Cl^−^ concentration (Cl_i_) of 30 mM was used assuming that Cl^−^ ions are at equilibrium at a resting potential of −40 mV. Since the permeability coefficients P_K_, P_Na_ and P_Cl_ of HEK cells are not known, a high P_Na_ model (P_K_ : P_Na_ : P_Cl_ = 1.00∶ 0.18∶ 0.10) and a low P_Na_ model (P_K_ : P_Na_ : P_Cl_ = 1.00∶ 0.10∶ 0.10) were used in the calculations. For the plot, mean ΔF/F_0_ and ion concentrations from the experiments with high K^+^ steps (Fig. 2D) were transformed according to eq. 12. Shown are the transformed data and fittings with z′ values of −1.48 and −1.04 for the high P_Na_ (green) and low P_Na_ (blue) models, respectively. (**B**) Test of the accuracy of z′ values determined with high K^+^ steps. Using mean ΔF/F_0_ values measured photometrically in voltage-clamped cells (Fig. 2F), ΔE was predicted with the z′ values that were determined by fitting the data of high K^+^ steps. The calculation of ΔE was performed following eq. 9. RT/F = 25.43 mV (22°C). The accuracy of z′ was tested by comparing the calculated ΔE with the experimentally applied voltage-clamp steps (ΔE_vc_). The graph depicts plots of ΔE vs. ΔE_vc_ with z′ values of −1.48 (green), −1.04 (blue) and −0.64 (black) as determined with high and low P_Na_ models (see above) and with the fraction K_x_/K_r_ (Fig. 4B), respectively. Since photometry combined with voltage clamp provides the most accurate measurement of the dye response to membrane potential changes, a plot of ΔE vs. ΔE_vc_ is shown for the z′ value of −0.71 (grey) that was obtained by fitting the voltage-clamp data (Fig. 4A). A comparison of ΔE vs. ΔE_vc_ plots demonstrates that the estimate of z′ based on the fraction K_x_/K_r_ closely resembles the measurement of z′ in voltage-clamped cells.
(TIF)
