# Supplementary figures and images for: Membrane Potential Measurements of Isolated Neurons Using a Voltage-Sensitive Dye
Source: PLoS One. 2013 Mar 13;8(3):e58260. doi: 10.1371/journal.pone.0058260 (PMC3596405; doi:10.1371/journal.pone.0058260)

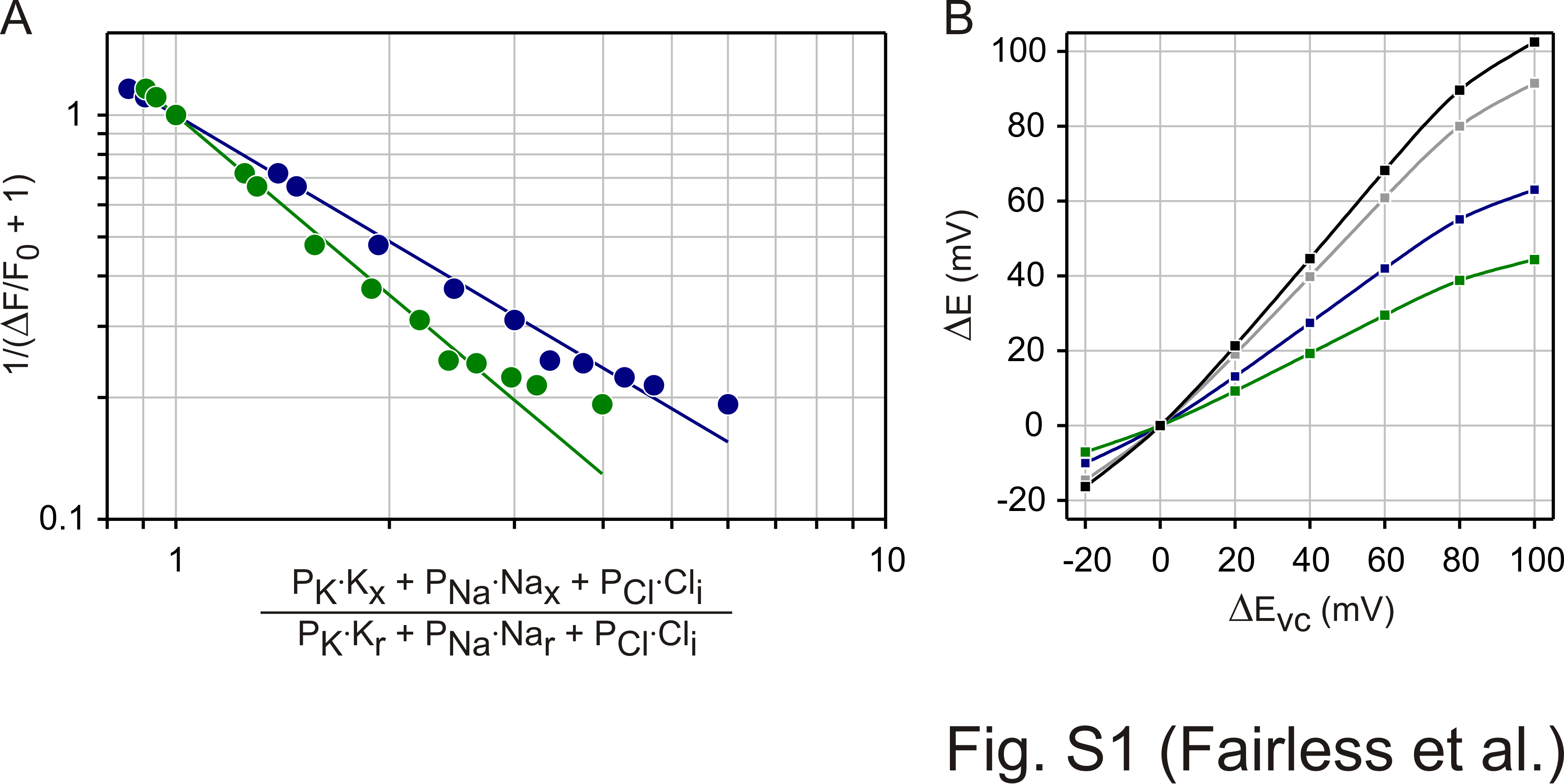

Supplement: Figure S1 — Influence of membrane ion permeabilities on calculations of the apparent charge (z′) of the FMP dye. (A) Determination of z′ considering the contribution of K+, Na+ and Cl− ions to changes in the membrane potential. An approach based on the Goldman-Hodgkin-Katz equation was used to estimate z′. Accordingly, the data obtained with high K+ steps (Fig. 2D) was fitted with eq. 12. Kx and Nax represent the changing external K+ and Na+ concentrations, respectively. In order to keep constant the osmolarity of high KCl solutions, Nax was reduced to maintain Kx+Nax = 150 mM for Kx>10 mM (see Methods). The reference concentrations Kr and Nar were 4 mM and 140 mM, respectively. An internal Cl− concentration (Cli) of 30 mM was used assuming that Cl− ions are at equilibrium at a resting potential of −40 mV. Since the permeability coefficients PK, PNa and PCl of HEK cells are not known, a high PNa model (PK : PNa : PCl = 1.00∶ 0.18∶ 0.10) and a low PNa model (PK : PNa : PCl = 1.00∶ 0.10∶ 0.10) were used in the calculations. For the plot, mean ΔF/F0 and ion concentrations from the experiments with high K+ steps (Fig. 2D) were transformed according to eq. 12. Shown are the transformed data and fittings with z′ values of −1.48 and −1.04 for the high PNa (green) and low PNa (blue) models, respectively. (B) Test of the accuracy of z′ values determined with high K+ steps. Using mean ΔF/F0 values measured photometrically in voltage-clamped cells (Fig. 2F), ΔE was predicted with the z′ values that were determined by fitting the data of high K+ steps. The calculation of ΔE was performed following eq. 9. RT/F = 25.43 mV (22°C). The accuracy of z′ was tested by comparing the calculated ΔE with the experimentally applied voltage-clamp steps (ΔEvc). The graph depicts plots of ΔE vs. ΔEvc with z′ values of −1.48 (green), −1.04 (blue) and −0.64 (black) as determined with high and low PNa models (see above) and with the fraction Kx/Kr (Fig. 4B), respectively. Since photometry combined with volta [file pone.0058260.s001.tif]
